# Genomic and microarray analysis of aromatics degradation in *Geobacter metallireducens *and comparison to a *Geobacter *isolate from a contaminated field site

**DOI:** 10.1186/1471-2164-8-180

**Published:** 2007-06-19

**Authors:** Jessica E Butler, Qiang He, Kelly P Nevin, Zhili He, Jizhong Zhou, Derek R Lovley

**Affiliations:** 1 Department of Microbiology, University of Massachusetts, Amherst, MA 01003, USA; 2 Environmental Science Division, Oak Ridge National Laboratory, Oak Ridge, TN 37831, USA; 3 Department of Civil and Environmental Engineering, University of Tennessee, Knoxville, TN 37996, USA

## Abstract

**Background:**

Groundwater and subsurface environments contaminated with aromatic compounds can be remediated *in situ *by *Geobacter *species that couple oxidation of these compounds to reduction of Fe(III)-oxides. *Geobacter metallireducens *metabolizes many aromatic compounds, but the enzymes involved are not well known.

**Results:**

The complete *G. metallireducens *genome contained a 300 kb island predicted to encode enzymes for the degradation of phenol, *p*-cresol, 4-hydroxybenzaldehyde, 4-hydroxybenzoate, benzyl alcohol, benzaldehyde, and benzoate. Toluene degradation genes were encoded in a separate region. None of these genes was found in closely related species that cannot degrade aromatic compounds. Abundant transposons and phage-like genes in the island suggest mobility, but nucleotide composition and lack of synteny with other species do not suggest a recent transfer. The inferred degradation pathways are similar to those in species that anaerobically oxidize aromatic compounds with nitrate as an electron acceptor. In these pathways the aromatic compounds are converted to benzoyl-CoA and then to 3-hydroxypimelyl-CoA. However, in *G. metallireducens *there were no genes for the energetically-expensive dearomatizing enzyme. Whole-genome changes in transcript levels were identified in cells oxidizing benzoate. These supported the predicted pathway, identified induced fatty-acid oxidation genes, and identified an apparent shift in the TCA cycle to a putative ATP-yielding succinyl-CoA synthase. Paralogs to several genes in the pathway were also induced, as were several putative molybdo-proteins. Comparison of the aromatics degradation pathway genes to the genome of an isolate from a contaminated field site showed very similar content, and suggested this strain degrades many of the same compounds. This strain also lacked a classical dearomatizing enzyme, but contained two copies of an eight-gene cluster encoding redox proteins that was 30-fold induced during benzoate oxidation.

**Conclusion:**

*G. metallireducens *appears to convert aromatic compounds to benzoyl-CoA, then to acetyl-CoA via fatty acid oxidation, and then to carbon dioxide via the TCA cycle. The enzyme responsible for dearomatizing the aromatic ring may be novel, and energetic investments at this step may be offset by a change in succinate metabolism. Analysis of a field isolate suggests that the pathways inferred for *G. metallireducens *may be applicable to modeling *in situ *bioremediation.

## Background

Some bacteria can metabolize the toxic aromatic compounds that are present in groundwater contaminated by petroleum or by landfill leachate [1-4]. The bacteria use these contaminants as the carbon and energy sources for growth, and they can oxidize many aromatic compounds completely to carbon dioxide. Both the species that accomplish this bioremediation and the mechanism with which they degrade these stable compounds are being investigated.

The benzene-ring structure of aromatic compounds must be dearomatized before they can be fully degraded. While oxygen can be used to attack the ring, many contaminated environments are anaerobic, so a different mechanism is needed [5]. The best-studied anaerobic species that catalyze this reaction couple the oxidation of aromatics to the reduction of high potential compounds like nitrate [4,6,7]. This metabolism provides enough energy to allow the dearomatization of the benzene-ring by an enzyme that uses two ATP and two low potential electrons instead of oxygen [8-10].

However, insoluble Fe(III)-oxides are the primary electron acceptor in many contaminated environments [11-14], and the energy gained from reducing Fe(III)-oxides (ΔE°' ≅ 200 mV) is less than that from nitrate (ΔE°' = 420 mV). It has been suggested that bacteria that use lower potential electron acceptors like Fe(III) or sulfate, or bacteria that ferment aromatic compounds, require a less energetically expensive mechanism to dearomatize the aromatic ring [15-17]. These enzymes have not yet been identified.

Molecular analyses have shown that members of the *Geobacter *family are the most abundant species in contaminated environments where Fe(III) is the electron acceptor [14,18,19]. *Geobacter metallireducens *was the first pure-culture organism found to anaerobically oxidize an aromatic hydrocarbon, toluene [12]. This species is also able to completely oxidize the aromatic compounds phenol, *p*-cresol, 4-hydroxybenzaldehyde, 4-hydroxybenzoate, benzyl alcohol, benzaldehyde, and benzoate, all with Fe(III) serving as the sole electron acceptor [20,21].

Two recent studies sought to identify enzymes involved in benzoate oxidation in *G. metallireducens*, specifically the enzyme that catalyzes the dearomatization of the benzene-ring [22,23]. Comparison of protein abundance in *G. metallireducens *growing by benzoate versus acetate oxidation identified 14 proteins that were more abundant during benzoate metabolism [23]. The genes encoding these proteins were located on two contigs in the partially completed *G. metallireducens *genome, and an additional 19 nearby genes were shown to have increased transcript levels in the presence of benzoate compared to acetate [23]. Several of the induced genes and proteins had clear sequence homology to those that catalyze benzoate metabolism in the nitrate-reducer *Thauera aromatica*, with one exception. No proteins similar to known benzoyl-CoA reductases, the enzyme that catalyzes the ring reduction, were found to have increased expression during benzoate oxidation, and no benzoyl-CoA reductase activity was detected in cell extracts [23].

A separate study suggests that genes for a benzoyl-CoA reductase were present elsewhere in the *G. metallireducens *genome [22]. Degenerate primers were designed from known benzoyl-CoA reductase α subunits, and used to amplified sequence from *G. metallireducens *genomic DNA [22]. Analysis of the corresponding gene and its flanking region led to the conclusion that the gene was the α subunit of the benzoyl-CoA reductase. The same analysis was done with a *Geobacter *species that cannot degrade aromatic compounds, and no such gene could be amplified [22].

The aim of this study is to use the completed genome sequence of *G. metallireducens *to predict the pathways for degradation of toluene, phenol, *p*-cresol, 4-hydroxybenzaldehyde, 4-hydroxybenzoate, benzyl alcohol, benzaldehyde, and benzoate. Support for the predicted pathways is provided by analysis of genome-wide changes in transcript levels by DNA microarray in cells oxidizing benzoate. The relevance of the *G. metallireducens *pathways to *in situ *environmental bioremediation is examined by comparison to a recently sequenced *Geobacter *isolate from a contaminated site.

## Results and discussion

### The 300 kb island of aromatics-degradation genes

The two gene clusters previously identified as encoding proteins and genes with increased expression during benzoate oxidation [23] were encoded ca. 60 kb apart in the completed genome (see Additional file 1) (accession NC_007517) [24]. These clusters were part of a 300 kb, 244-gene region (Gmet_2037-Gmet_2284) in which very few of the encoded proteins had orthologs in the genome of *G. sulfurreducens*, a closely related *Geobacter *species that cannot metabolize aromatic compounds (see Additional file 1). Orthologs were defined as proteins that were reciprocal best matches when all proteins from both genomes were aligned, with a minimum alignment of 60% of both proteins. Across the whole *G. metallireducens *genome, 2130 proteins (60.3%) had orthologs in *G. sulfurreducens*, but in this region of the genome only 20 (8%) had orthologs in the *G. sulfurreducens *genome (see Additional file 1).

When the 244 proteins encoded in this region were aligned against the entire non-redundant protein database from NCBI, many had highest sequence similarity to enzymes involved in aromatics degradation in the nitrate-reducing organisms *Thauera aromatica *and *Azoarcus *sp. EbN1 (see Additional file 1). However, the overall organization of the genes in the island was not found to be conserved when compared to any other organism. Analysis of the composition and structure of this region is discussed below.

### Phenol, *p*-cresol, 4-hydroxybenzaldehyde, 4-hydroxybenzoate, benzyl alcohol, and benzaldehyde metabolism

**Figure 1:**
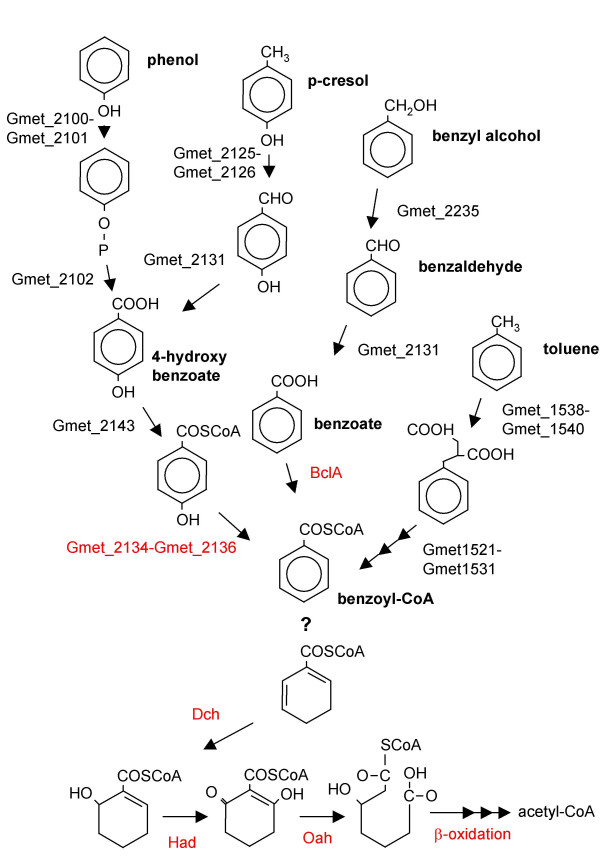
**Pathways of oxidation of aromatic compounds in *G. metallireducens *inferred from the whole-genome gene content**. Enzymes of the benzoyl-CoA pathway indicated: benzoate CoA ligase (BclA), cyclohexa-1,5-diene-1-carbonyl-CoA hydratase (Dch), 6-hydroxycylohex-1-en-1-carbonyl-CoA dehydrogenase (Had), 6-oxocyclohex-1-ene-1-carbonyl-CoA hydratase (Oah). Red coloring indicates that the gene was induced during growth by benzoate oxidation. The "?" indicates that no orthologs to known benzoyl-CoA reductases found. Figure adapted from [7].

During phenol metabolism in *T. aromatica*, a two-subunit phenylphosphate synthase and a phenylphosphate carboxylase convert phenol to 4-hydroxybenxoate [25-27]. In *G. metallireducens*, homologs to these genes were found in the aromatics region, in a putative operon (Gmet_2100-Gmet_2102) (Figure 1, Additional file 1).

In previously studied bacteria, *p*-cresol is metabolized by conversion to 4-hydroxybenzoate via 4-hydroxybenzaldehyde. A flavocytochrome *p*-cresol methylhydroxylase creates 4-hydroxybenzaldehyde, which is then oxidized via a 4-hydroxybenzaldehyde dehydrogenase [28,29]. In *G. metallireducens*, homologs to the two-subunit methylhydroxylase (Gmet_2125-Gmet_2126) are encoded just upstream of the putative dehydrogenase (Gmet_2131), all within the aromatics island of the genome (Figure 1, Additional file 1). These genes have high sequence similarity to those in *Azoarcus*-related species and *Pseudomonas putida *[28,29].

In *T. aromatica*, 4-hydroxybenzoate, benzyl alcohol, and benzaldehyde are all metabolized to the central intermediate benzoyl-CoA. 4-hydroxybenzoate is converted via a Co-A ligase and a well-studied reductase [30]. In *G. metallireducens*, a three-gene cluster in the aromatics island of the genome (Gmet_2134-Gmet_2136) had identical organization and high sequence similarity to the 4-hydroxybenzoyl-CoA reductase operon in *T. aromatica *(Figure 1, Additional file 1). In *G. metallireducens*, the dehydrogenase genes predicted to be responsible for the conversion of benzyl alcohol and benzaldehyde to benzoate (Gmet_2235 and Gmet_2131) have closest homologs to those enzymes in *Azoarcus*-related species and *Pseudomonas *species (Figure 1, Additional file 1) [29,31]. The specificity of the CoA ligases for 4-hydroxybenzoate and benzoate is difficult to predict based on sequence identity alone. However, the benzoate CoA ligase (BclA) was previously identified and characterized [23], and in the completed genome is encoded by Gmet_2143 (Figure 1, Additional file 1).

### The putative benzoyl-CoA reductase

The pathways that can be inferred from the analysis presented above predict that, like nitrate-reducing species, all of the aromatic compounds that *G. metallireducens *is capable of degrading are converted to the central metabolite benzoyl-CoA (Figure 1). However, benzoyl-CoA is still aromatic, and dearomatization of the benzene-ring structure is the central step in the degradation pathways. The enzyme that catalyzes this reaction in nitrate-reducing species, the benzoyl-CoA reductase, has been well studied [5,9]. Previously, a gene predicted to encode this enzyme in *G. metallireducens *was identified (NCBI accession YP_383512) [22], and in the completed genome, this gene was encoded by Gmet_0544. This gene was not located in the aromatics island of the genome.

Examination of this gene, its closest phylogenetic relatives, and the adjacent genes indicate that this gene does not appear to be part of a classical benzoyl-CoA reductase. Gmet_0544 had highest amino acid identity to the ATP-binding activator subunit of 2-hydroxyglutaryl-CoA dehydratases, an enzyme involved in the fermentation of glutamate via the hydroxyglutarate pathway [32]. Gmet_0544 was more similar to the characterized activator subunits from *Clostridia symbiosum *[33] and *Acidaminococcus fermentans *[34] (ca. 30%), than to the benzoyl-CoA reductase proteins from *T. aromatica*, *Azoarcus *species, or *R. palustris *(ca. 17%).

**Figure 2:**
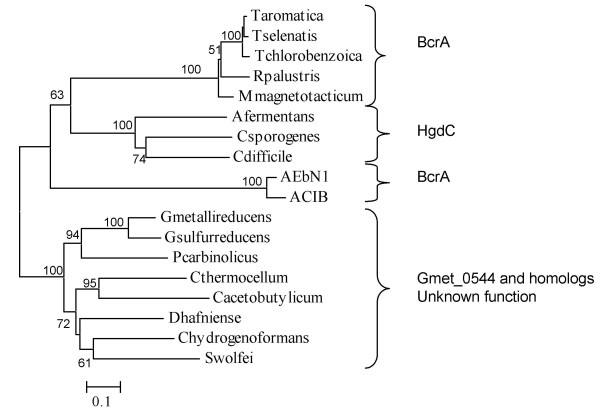
**Phylogeny of characterized benzoyl-CoA reductase α subunit genes (BcrA)**. Also shown are characterized 2-hydroxyglutaryl-CoA dehydratase α subunit genes (HgdC), and homologs including Gmet_0544. Proteins were aligned with ClustalX, and distance and branching order were determined by the Neighbor Joining method with the Poisson correction. 1000 bootstrap replicates were used, and values above 50% are shown.

A phylogenetic tree was constructed for Gmet_0544 and the activator subunits of known benzoyl-CoA reductases and 2-hydroxyglutaryl-CoA dehydratases (Figure 2). The benzoyl-CoA reductases clustered in two groups, the *Thauera*-species group and the *Azoarcus*-species group, as has been previously described (Figure 2) [7]. As expected, characterized 2-hydroxyglutaryl-CoA dehydratases subunits from *Clostridia *species also formed a distinct clade (Figure 2). The protein encoded by Gmet_0544 clustered with homologous proteins from two other *Geobacteraceae *species that do not oxidize aromatic compounds, and with a variety of uncharacterized proteins from *Clostridia *and other species (Figure 2).

**Figure 3:**
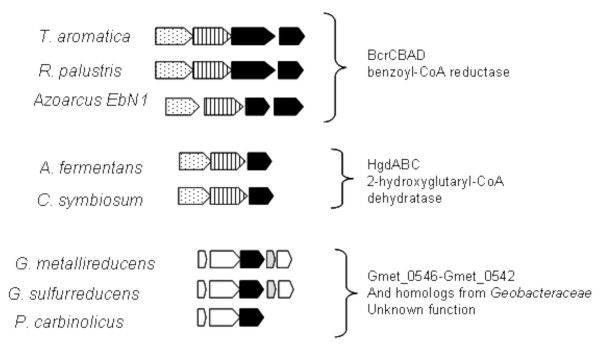
**Operons for benzoyl-CoA reductases, 2-hydroxyglutaryl-CoA dehydratases, and the region of *G. metallireducens *genome containing Gmet_0544**. Gmet_0544 was previously identified benzoyl-CoA reductase α subunit [22]. Also shown are the regions in the non-aromatics degrading species *G. sulfurreducens *and *P. carbinolicus *that contain the Gmet_0544 orthologs. Black – activase subunits, Striped – catalytic subunit, Dotted – catalytic subunit, White – conserved hypothetical, Grey – potential cytochrome.

More importantly, Gmet_0544 is not a likely part of either a functional 2-hydroxyglutaryl-CoA dehydratase or a functional benzoyl-CoA reductase, because there were no genes in the genome that were predicted to encode the catalytic subunits of these enzymes. Sequences of the four subunit benzoyl-CoA reductases and of the three subunit 2-hydroxyglutaryl-CoA dehydratases were compared with all proteins in the completed *G. metallireducens *genome (Figure 3). There was no cluster, either inside the aromatics island or elsewhere, that resembled these enzymes. The two ATP-binding subunits of these enzymes both had some similarity to Gmet_0544 (Figure 3, Table 1). However, the catalytic subunits had no significant matches in the *G. metallireducens *genome (Figure 3, Table 1).

**Table 1:** *G. metallireducens *proteins most similar to the benzoyl-CoA pathway proteins from *T. aromatica*^a^

Benzoyl-CoA pathway protein	closest Gmet ORF	% a.a. ID	name
BclA	Gmet_2143	19	BamY*
BcrA	Gmet_0544	50	Bcra#
BcrB	none		
BcrC	none		
BcrD	Gmet_0544	24	Bcra#
Fdx	Gmet_1033	22	
Dch	Gmet_2150	73	BamR*
Had	Gmet_2151	70	BamQ*
Oah	Gmet_2088	76	BamA*

^a ^Enzymes are benzoate CoA ligase (BclA), benzoyl-CoA reductase (BcrCDAB), ferredoxin (Fdx), cyclohexa-1,5-diene-1-carbonyl-CoA hydratase (Dch), 6-hydroxycylohex-1-en-1-carbonyl-CoA dehydrogenase (Had), and 6-oxocyclohex-1-ene-1-carbonyl-CoA hydratase (Oah).

* Gene identified and named by Wishgoll. # Gene identified and named by Hasoda. The cut-off value used for a significant match in the *G. metallireducens *genome was 0.1.

### Metabolism of the non-aromatic products

In other anaerobic aromatics degraders the reduction of benzoyl-CoA produces cyclohexa-1,5-diene-1-carbonyl-CoA, which is then converted to 3-hydroxypimelyl-CoA [35]. In *G. metallireducens*, homologs to the proteins that catalyze this conversion are expressed during benzoate oxidation [23]. These genes, the cyclohexa-1,5-diene-1-carbonyl-CoA hydratase (*dch*, Gmet_2150), 6-hydroxycylohex-1-en-1-carbonyl-CoA dehydrogenase (*had*, Gmet_2151), and 6-oxocyclohex-1-ene-1-carbonyl-CoA hydratase (*oah*, Gmet_2088), are all located in the aromatics region of the genome (Table 1).

The 3-hydroxypimelyl-CoA that results from this pathway enters into the beta fatty acid oxidation pathway, where it is converted to acetyl-CoA [7]. In *G. metallireducens*, there are several clusters in the aromatics island that encode genes predicted to be involved in fatty acid oxidation, including Gmet_2057-Gmet_2075, Gmet_2194-Gmet_2213, and Gmet_2268-Gmet_2270. Putative acyl-CoA dehydrogenases, enoyl-CoA hydratases, thiolases, and Co-A transferases were all present in multiple copies with high sequence similarities (see Additional file 1). However, unlike the other genes predicted to be involved in aromatics degradation in *G. metallireducens*, the closest homologs in the NCBI database are not from known aromatics degraders. Closest homologs to these genes were found in a variety of species including *Clostridia*, *gamma Proteobacteria*, and *Actinobacteria *(see Additional file 1).

Thus, while *G. metallireducens *lacks the central enzyme of the pathway, the ATP-dependent benzoyl-CoA reductase, it contains genes for the oxidation of the product of benzoyl-CoA reduction, including *dch*, *had*, *oah*, and fatty acid pathway genes (Figure 1).

### Paralogs of the enzymes BamB, BamC, and Oah

It has been suggested that an uncharacterized protein called BamB (Gmet_2087) could be a possible alternative to the benzoyl-CoA reductase, because it was shown to be more abundant in benzoate-grown cells of *G. metallireducens *than cells grown on acetate [23]. Analysis of the completed genome showed that there was a second protein with homology to BamB encoded outside of the aromatics island of the genome. Gmet_1802 encoded a protein of identical length to Gmet_2087, with 66% amino acid identity. This gene was encoded in a small region that lacked orthologs in *G. sulfurreducens *(Gmet_1784-Gmet_1815). This cluster also contained a close homolog to the putative Fe-S protein that is encoded upstream of *bamB*, called BamC. BamC and its paralogs (Gmet_1803) have 64% amino acid identity to each other.

**Figure 4:**
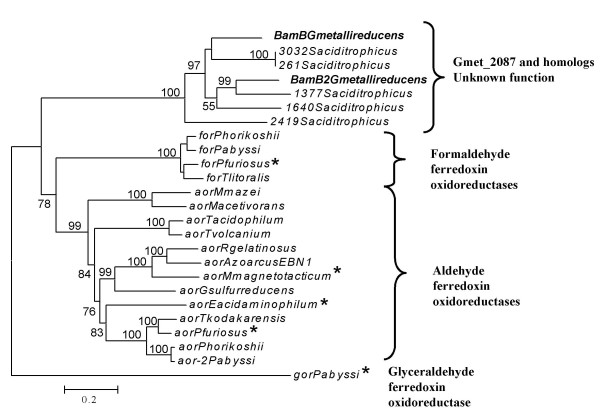
**Phylogenetic tree inferred for characterized tungsten-containing ferredoxin oxidoreductases and the BamB homologs Gmet_2087 and Gmet_1802**. Functional annotation for the clades was based on the characterized proteins (starred). Distance and branching order were determined by the neighbor-joining method. 100 bootstrap replicates were used, and values above 50 are shown.

The function of BamB and its paralog cannot be readily deduced from their sequence. Of characterized proteins, they are most similar (ca. 25% amino acid identity) to formaldehyde and aldehyde ferredoxin oxidoreductases from *Archaea *species [36,37]. However, alignment of BamB and its homolog to these enzymes from *Pyrococcus furiosus *showed that, while the overall alignment is good, several important residues are not conserved. The residues that line the substrate pockets, the residues closest to the tungsten, and the residues that interact with the tungstopterin are not conserved. Phylogenetic analysis confirmed that the *G. metallireducens *proteins were clearly distinct from the clades of formaldehyde, aldehyde, and glyceraldehyde ferredoxin oxidoreductases (Figure 4). None of the characterized tungsto-enzymes grouped with the *G. metallireducens *enzymes, but homologs were found in *S. aciditrophicus*, a benzoate fermenter (Figure 4) [38].

A second enzyme predicted to be involved in the central part of the pathway of benzoate oxidation in *G. metallireducens *was also found to have a homologous gene outside of the aromatics region of the genome. Gmet_3305, with 86% amino acid identity to Oah (Gmet_2088) in the aromatics island, was found distant in the genome in a 30-gene region that lacked orthologs in *G. sulfurreducens *(Gmet_3280-Gmet_3309).

The organization and duplication of these central genes in the benzoyl-CoA pathway are unique to *G. metallireducens*. In *T. aromatica*, *Azoarcus *species, *R. palustris*, and *Magnetospirillum magnetotacticum*, the central genes in the pathway (the benzoyl-CoA reductase, Dch, Had, and Oah) are always encoded in a large cluster [7,39,40]. *G. metallireducens *lacks the reductase, and the roles of BamB and its newly identified homolog cannot be predicted based on sequence analysis alone. The *dch *and *had *genes are adjacent, but neither is near either of the two very similar *oah *homologs.

### The toluene degradation genes

In addition to the aromatics compounds described above, *G. metallireducens *can also degrade the hydrocarbon toluene [12]. The genes in *G. metallireducens *are similar to those for conversion of toluene to benzoyl-CoA in other organisms, and have been described previously (Figure 1) [41]. There are two apparent operons encoded next to each other, for the benzylsuccinate synthase (*bssCAB*, Gmet_1538-Gmet_1540) and for benzylsuccinate oxidation to benzyl-CoA (*bbsABCD*, Gmet_1528-1531, and *bbsEFGH*, Gmet_1521-Gmet_1524). In the completed genome, these genes were not located near the other aromatics degradation genes. Instead, they were encoded 556 kb upstream, in a region that, like the large island, lacked orthologs in *G. sulfurreducens*. In this 167 kb region, Gmet_1435-Gmet_1572, only 25 of 133 genes (19%) had orthologs in the *G. sulfurreducens *genome.

### Characterization of the large genomic island

Genes for aromatics degradation have been found as parts of catabolic transposons [42]. In an effort to understand the origin of the aromatics degradation genes in *G. metallireducens*, analysis of local compositional heterogeneity and identification of potential mobile genetic elements were performed [43,44].

**Figure 5:**
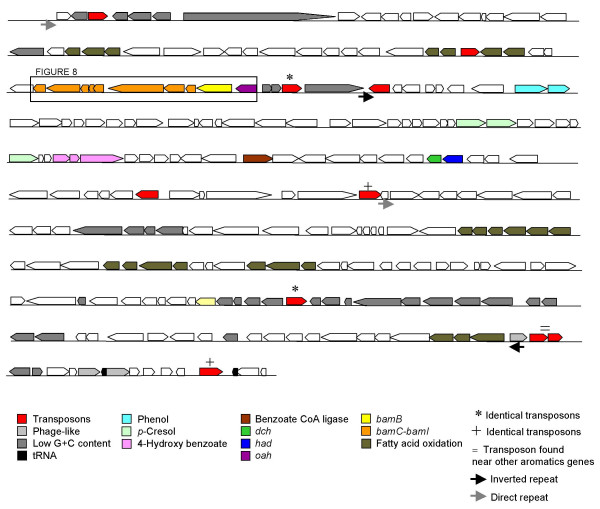
**Schematic of the 300 kb aromatics island in the genome (Gmet_2037-Gmet_2284)**. Predicted functions of genes are shown. Only those genes predicted to be involved in aromatics metabolism are shown in color. Repetitive regions are shown with matching symbols above the region. Low GC content genes are stripped. The region represented in Figure 8 is shown in the rectangle.

**Figure 6:**
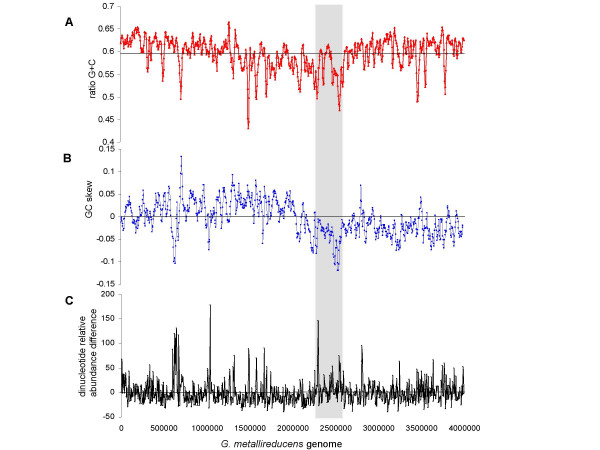
**The nucleotide composition of the whole genome**. A) The GC content plotted for windows of 20 kb with a 5 kb step. Horizontal black line indicates GC content average for the genome (59.5 %). B) The GC skew plotted for windows of 20 kb with a 5 kb step. C) The dinucleotide relative abundance difference with respect to the whole genome average, calculated using delta-rho with a non-overlapping window of 5 kb [71]. The scale is identical in all plots. The aromatics island is shown by the gray box.

The genome average for GC content was 59.5%. The start of the large aromatic island was defined by a region of significantly atypical composition. The 5' end (near base 2,280,000) had low GC content and GC skew, and an atypical dinucleotide relative abundance (Figure 5, Figure 6). This point was also the beginning of the region that had very low ortholog frequency compared to the *G. sulfurreducens *genome (see Additional file 1). There was another small region of low GC content that separated *oah *and the *bam *cluster of genes from the other genes predicted to be involved in aromatics metabolism (Figure 5, Figure 6). However, neither the *bam *cluster nor any of the genes predicted to be involved in aromatics metabolism (Figure 1) had significant deviance in composition compared to the rest of the genome (Figure 5, Additional file 1). The last third of large island had atypically low GC content and atypical dinucleotide relative abundance (Figure 5, Figure 6, Additional file 1). The 3' end of the island was defined as the point near base 2,590,000 that coincided with the end of the low GC region and the end of the region that lacked orthologs to *G. sulfurreducens *(Figure 5, Figure 6, Additional file 1).

### Transposons and repetitive sequences

There were 9 transposase insertion sequence elements flanked with perfect inverted repeats (IS) in the large aromatics island (Figure 5). At the 3' end of the large aromatics island was a putative two gene transposase operon, Gmet_2272-Gmet_2273, of the *istAB*-type [45]. Exact copies of this pair, with 100% nucleic acid identity including the flanking inverted repeats, were found in two other places in the genome: upstream of the *bamB *homolog (Gmet_1788-Gmet_1789), and upstream of the *oah *homolog (Gmet_3281-Gmet_3282).

The eight other IS elements in the large aromatics island were Gmet_2039, Gmet_2073, Gmet_2091, Gmet_2093, Gmet_2160, Gmet_2166, Gmet_2240, and Gmet_2284 (Figure 5). Based on sequence similarity, these transposons belong to a variety of families, including IS4, IS5, ISl3, IS21, and IS110 [45,46]. Two pairs of these IS elements had 100% nucleic acid identity to each other (Gmet_2091 to Gmet_2240 and Gmet_2166 to Gmet_2284). These identical pairs could form a larger, composite transposon that would include all the intervening genes (Figure 5).

There was a 131 bp direct repeat in the aromatics island, with the left copy at the 5' end of the island between Gmet_2036 and Gmet_2037 (coordinates 2280680–2280810), and the right copy between Gmet_2166 and Gmet_2168 (Figure 5). There was also a 130 bp inverted repeat, with the left copy between Gmet_2092 and Gmet_2093 (coordinates 2280680–2280810), and the right copy within Gmet_2271 (Figure 5).

### Phage-like genes

All four regions of aromatics metabolism genes were flanked on at least one side by tRNA genes. tRNA genes are often associated with genomic islands [47], and are often target sites for phage attachment and integration [48]. The 3' end of the large aromatics island was flanked by a tRNA-Gly gene, and a pseudo tRNA was found 6.6 kb upstream (Figure 5). Three genes with homology to phage integrases were encoded nearby (Gmet_2271, Gmet_2278, Gmet_2279) (Figure 5).

There was a tRNA-Gly near the *bamB *homolog (Gmet_1802), as well as a putative phage integrase (Gmet_1783). The region that contained the *oah *homolog had a tRNA-Ala at the 5' end. The toluene region was flanked on both sides by tRNA genes: a tRNA-Met at the 5' end and a tRNA-Val at the 3' end.

Thus, the 300 kb region that contains the predicted aromatics degradation genes shows many of the hallmarks of genomic islands [47]. The genes in these regions are species-specific; they have no orthologs in the closely related species, *G. sulfurreducens*, which cannot degrade aromatics. The genes are encoded in large, discrete units, flanked by genes with atypical nucleotide composition, tRNA genes, and phage-like integrases. The region shows signs of potential genetic mobility, including identical transposons and repetitive sequences. However, the genes predicted to be involved in aromatics metabolism do not have atypical composition compared to the rest of the genome, and they lack synteny with the genes from other aromatics degraders. This implies that if there were horizontal gene transfer event(s) from distantly related organisms, the genes have since ameliorated [43,49].

### Gene expression changes during benzoate metabolism

**Figure 7:**
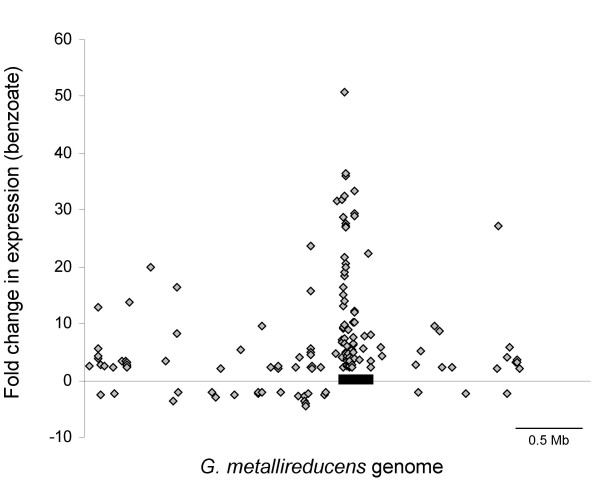
**Locations in genome of genes determined by microarray analysis to have changes in transcript abundance**. mRNA content from cells using benzoate vs. acetate as the sole electron and carbon source was compared, with soluble Fe(III) as the terminal electron acceptor in both cases. Distance from the horizontal line represents fold change in transcript levels, with only those genes with a two-fold or greater change and a significance of p ≤ 0.01 shown. The thick black bar represents the large aromatics island Gmet_2037-Gmet_2284.

In order to further evaluate the genes predicted to be involved in the degradation of aromatic compounds, changes in levels of gene transcripts were compared for 3417 ORFs (97% of the genome) with microarray analysis (NCBI Gene Expression Omnibus, accession GSE5401). Cells were grown in continuous-culture chemostats with either benzoate or acetate as the limiting electron donor and with Fe(III) as the electron acceptor. A total of 121 genes (3.4% of the genome) had transcript levels that were at least 2-fold higher during growth with benzoate (see Additional file 2). Analysis of the distribution of these genes across the genome showed that the majority (64) were encoded in the 300 kb aromatics island (Figure 7). 36 genes (1.0% of the genome) had at least 10-fold higher transcript levels during benzoate oxidation (Table 2). Most of these genes (28) were in the aromatics island of the genome. A total of 21 genes (0.6 % of the genome) had at least 2-fold decreased expression levels during benzoate oxidation, with a maximum decrease of 4.6 fold (see Additional file 2). None of these down-regulated genes were located in the aromatics island of the genome (Figure 7).

**Table 2:** *G. metallireducens *genes for which transcription was induced at least 10-fold with benzoate as the sole electron and carbon donor, relative to acetate

Gmet ORF^a^	Fold change	Gene name	Predicted function
Gmet_0108	12.9		ATPase
Gmet_0356	13.8		Conserved hypothetical protein
Gmet_0519	20		Permease
Gmet_0728	16.5		Conserved hypothetical protein
Gmet_1802	23.6		Aldehyde ferredoxin oxidoreductase
Gmet_1803	15.7		4Fe-4S binding
Gmet_2017	31.5		Hypothetical protein
**Gmet_2057**	31.9	ech^	Enoyl-CoA hydratase/isomerase
**Gmet_2058**	15.2	act*	Thiolase
**Gmet_2059**	16.4	adh	Short-chain dehydrogenase
**Gmet_2064**	13.2	tre^	Bacterial regulatory proteins, IclR
**Gmet_2065**	28.7	oxr	4Fe-4S binding
**Gmet_2068**	32.6	scsA*	Succinyl-CoA synthetase, alpha
**Gmet_2071**	14.1	ech*	Enoyl-CoA hydratase/isomerase
**Gmet_2072**	18.4	acd^	3-hydroxyacyl-CoA dehydrogenase
**Gmet_2074**	50.7	bamN^	Thiolase
**Gmet_2075**	19.1	bamM*	Acyl-CoA dehydrogenase
**Gmet_2077**	21.8	bamK	N-acetyltransferase
**Gmet_2080**	36.1	bamH^	NAD(P) diaphorase, HoxF
**Gmet_2081**	27.5	bamG	NAD(P) diaphorase, HoxU
**Gmet_2083**	27.3	bamF^	hydrogenase, delta subunit; HTH mo
**Gmet_2084**	20.6	bamE*	4Fe-4S binding, FAD binding
**Gmet_2085**	27	bamD^	4Fe-4S binding, cysteine rich
**Gmet_2086**	20	bamC*	4Fe-4S binding
**Gmet_2087**	36.4	bamB*	Aldehyde ferredoxin oxidoreductase
**Gmet_2147**	10.4	bamU^	Amidohydrolase
**Gmet_2149**	12.1	bamS	Hypothetical
**Gmet_2150**	33.4	bamR*	Dch
**Gmet_2151**	29.3	bamQ*	Had
**Gmet_2152**	28.9	bamP*	Electron transfer flavoprotein, alpha
**Gmet_2153**	10.2	bamO^	Electron transfer flavoprotein, beta
**Gmet_2157**	12		Response regulator receiver
Gmet_2260	22.4		Succinyl-CoA synthetase, alpha sub
Gmet_3305	27.2		Oah2

^a ^Genes in bold are located in the aromatics island of the genome.

* Genes encoding proteins with increased abundance during benzoate oxidation (Wischgoll et al., 2005).

^ Genes expressed during benzoate but not acetate oxidation (Wischgoll et al., 2005).

### The *Thauera*-like benzoate pathway

Previously, 14 proteins were identified as having increased expression during benzoate oxidation in *G. metallireducens *[23]. The microarray analysis identified increased transcript abundance in 13 of the genes that encode these proteins (see Additional file 2). 10 of these were among the very few ORFs in the genome that showed an increase of at least 10-fold during benzoate oxidation (Table 2).

The *dch *and *had *genes (Gmet_2150, Gmet_2151) were among the most strongly induced of all genes, with about 30-fold increase during benzoate oxidation (Figure 1, Table 2). Because the array was designed based on a draft version of the genome, a probe was created for only one of the two *oah *homologs, Gmet_3305. Like *dch *and *had*, this gene was found to be among the most strongly induced genes during benzoate oxidation, with a 27 fold change in transcript levels (Figure 1, Table 2). The other homolog to *oah*, Gmet_2088, has previously been shown to have increased expression during benzoate oxidation [23]. Thus, it appears that both *oah *homologs have increased expression during benzoate oxidation.

The induced proteins [23] were encoded in two small clusters inside the aromatics island, and an additional 19 genes within these clusters have been shown by RT-PCR to be induced by benzoate oxidation [23]. Microarray analysis identified 14 of these as induced during benzoate oxidation (see Additional file 2). In addition, microarray analysis also identified increases in transcript levels in several other genes in these two clusters that were not previously identified (see Additional file 2). Like the 13 genes identified by protein analysis, most of these genes were also at least 10-fold more abundant in benzoate oxidizing cells (Table 2). Transcripts for the benzoate-CoA ligase (Gmet_2143) had a smaller increase in abundance, 4.9 fold, than the other genes predicted to be involved in the benzoate oxidation pathway, (Figure 1, Additional file 2). Thus, microarray analysis of transcript abundance changes during benzoate oxidation reliably identified the genes predicted to be involved in benzoate oxidation (Figure 1).

### Gmet_0544

Unlike the other genes in the predicted benzoyl-CoA pathway, the gene previously identified as the α subunit of the benzoyl-CoA reductase (Gmet_0544) [22] had no change in abundance during benzoate oxidation (see Additional file 2). Neither did any of the neighboring genes (see Additional file 2). This result supports the analysis presented earlier that shows that this gene is not likely to be part of the pathway of aromatics degradation, and is not the benzoyl-CoA reductase.

### bamB and bamC homologs

Analysis of whole genome transcript changes during growth with benzoate showed that *bamB *had the second highest increase, 36.4-fold, of all genes (Table 2). In addition, the cluster of seven genes (Gmet_2080-Gmet_2087) that *bamB *was a part of was the most highly up-regulated cluster in the genome, ca. 25-fold (Figure 7, Table 2). As described above, a homolog to *bamB *was found outside the aromatics island of the genome. This homolog, Gmet_1802, also had a large increase in transcript levels, 23.6-fold, during growth on benzoate (Table 2). The cluster that contained *bamB2 *(Gmet_1795-Gmet_1810) was the only large gene cluster outside of the aromatics region that had a significant increase in transcript levels during growth on benzoate (see Additional file 2). Directly upstream of both *bamB *homologs were the homologous genes, *bamC *and *bamC2*, both encoding putative 4Fe-4S cluster proteins (Gmet_2086 and Gmet_1803). These genes were also induced more than 15-fold in the presence of benzoate (Table 2). Thus, like the two *oah *homologs, the two *bamB *and *bamC *homologs both have large increases in expression during growth by benzoate oxidation.

### Fatty acid oxidation

The completed genome of *G. metallireducens *contained several copies of genes predicted to be involved in fatty acid oxidation. Only one set, Gmet_2057-Gmet_2075, had marked increases in abundance during benzoate oxidation (see Additional file 2). This set included the gene with the greatest increase in transcript levels during growth on benzoate, a 50-fold induced thiolase (Gmet_2074) (Table 2). Also induced more than 15-fold were another thiolase (Gmet_2058), two acyl-CoA dehydrogenases (Gmet_2072, Gmet_2075), and two enoyl-CoA hydratases (Gmet_2057, Gmet_2071) (Table 2). Thus, though there are several homologs to beta fatty acid oxidation genes in the genome, only one cluster showed increases in expression during benzoate oxidation (Figure 5).

### Changes in the TCA cycle

The oxidation of one benzoate molecule via the benzoyl-CoA and fatty acid pathways results in three molecules of acetyl-CoA [7]. Acetate, another electron donor used by *Geobacter *species, is also converted to acetyl-CoA. In *G. sulfurreducens*, acetyl-CoA is oxidized to carbon dioxide via TCA cycle reactions, and in *G. metallireducens *most of the TCA cycle enzymatic activities have also been identified [50,51].

During growth with acetate as the electron donor, the TCA cycle of *G. sulfurreducens *does not include an ATP-yielding succinyl-CoA synthetase [51]. In other organisms, this enzyme catalyzes the conversion of succinyl-CoA to succinate and produces the only substrate-level phosphorylation of the TCA cycle. Instead, in *Geobacter *species, an acetate:succinyl-CoA transferase is used to convert succinyl-CoA to succinate coupled to the activation of acetate to acetyl-CoA [51]. Thus, there is no substrate-level ATP generation during acetyl-CoA oxidation.

However, analysis of the genome of *G. metallireducens *showed that there were three sets of genes predicted to encode the two-subunit ATP-yielding succinyl-CoA synthetase enzyme: Gmet_0729-Gmet_0730, Gmet_2068-Gmet_2069, and Gmet_2260-Gmet_2261. The latter two pairs were found in the aromatics island of the genome and shared 92% sequence identity (Supplementary Table 1). The third set of genes, Gmet_0729-Gmet_0730, was only ca. 55% identical to the pairs inside the aromatics island. At least one of the subunits of each of these pairs was shown to have increased transcript abundance during benzoate oxidation, with increases of 8 to 32 fold (see Additional file 2).

The ortholog in *G. metallireducens *to the acetate:succinyl-CoA transferase of *G. sulfurreducens *was encoded by Gmet_3044. This gene was one of the relatively few genes that had decreased abundance during benzoate oxidation, with a 2.2 fold decrease (see Additional file 2). When considered together, these two results – the decrease in the transferase gene and the increase in the synthetase genes – suggest that the enzyme that catalyzes the conversion of succinyl-CoA to succinate is changed when benzoate is the electron donor. Since the product of benzoate oxidation is likely acetyl-CoA and not acetate, there would be no need for an acetate:succinyl-CoA transferase. The exclusive use of a succinyl-CoA synthetase instead would allow for the generation of an ATP by substrate level phosphorylation for each of the three acetyl-CoA molecules predicted to be produced from benzoate oxidation.

### Molyboenzymes

Molybdenum has been shown to be required for benzoate oxidation in the sulfate-reducer *D. multivorans*, but is not required for nitrate-reducers [16]. A recent study showed only a slight decrease in growth in *G. metallireducens *cultured without molybdenum supplementation, but these results were qualified due to molybdenum impurities in the growth media [23].

Several putative molybo-proteins had higher transcript levels during growth on benzoate in *G. metallireducens *(see Additional file 2). As described above, both of the BamB homologs (Gmet_2087 and Gmet_1802) are related to tungsto- and molybo-proteins, and both had ca. 30-fold induction during benzoate oxidation. Nearby *bamB2 *were genes for a putative molybdenum cofactor biosynthesis protein (Gmet_1804) with 4.5-fold higher transcript level during growth on benzoate, and for a putative molybdopterin oxidoreductase, Gmet_1810, with 2.0-fold induction (see Additional file 2). Though no 4-hydroxybenzoate was supplied to the cultures, the genes for the molybdo-enzyme 4-hydroxybenzoate reductase (Gmet_2134-Gmet_2136) had ca. 2.5-fold transcript levels during growth on benzoate (see Additional file 2). In addition, a putative nitrate reductase operon, also a molybdo-enzyme (Gmet_0329-Gmet_0334) had ca. 3-fold higher transcript levels during growth on benzoate, though no nitrate was supplied (see Additional file 2).

### Down-regulated genes

As mentioned above, the enzyme predicted to be required for acetate activation, an acetate-CoA transferase was down-regulated 2.2 fold in the absence of acetate (see Additional file 2). Two apparent operons were also down-regulated: Gmet_1380-Gmet_1381, encoding proteins similar to those involved in disulfide bond formation, and Gmet_1753-Gmet_1756, encoding proteins similar to the glutamate synthases found in methanogens (see Additional file 2). Two putative transcriptional regulators were down-regulated, one of which shows similarity to the IclR family of regulators involved in acetate metabolism in *Escherichia coli *[52]. Also down-regulated were eight hypothetical or conserved hypothetical proteins (see Additional file 2).

### Genes in a *Geobacter *species from an aromatics-contaminated field site

A strain of *Geobacter *was recently isolated from sediments from the Department of Energy's Field Research Center in Oak Ridge, Tennessee (*Geobacter *strain FRC-32, Joel Kostka, personal communication). This site is contaminated with uranium, metals, and fuel hydrocarbons including toluene, and bioremediation by *in situ *bacteria is being studied there. *Geobacter *species have been shown to be present in sediments from the site [53,54], and the *Geobacter *population has been shown to increase by two orders of magnitude during stimulation of Fe(III) and uranium reduction [54]. The draft sequence of the *Geobacter *strain FRC-32 genome has recently been released (NCBI Accession number AASH00000000). The sequence of the 16S rRNA gene of *Geobacter *strain FRC-32 is 99.4 – 99.8 % identical to the *Geobacter *16S rRNA gene sequences cloned from the contaminated site [54]. The genome is 4.0 Mb, with 3396 open reading frames in 164 contigs, each with sequence coverage of at least 10×.

To evaluate the possibility of extrapolating the predicted pathways of aromatics degradation in *G. metallireducens *to an environment of bioremediation, the genes from *G. metallireducens *were compared to those encoded in the *Geobacter *strain FRC-32 genome. *Geobacter *strain FRC-32 encoded proteins with high sequence similarity and orthology to those in *G. metallireducens *for the oxidation of toluene, phenol, *p*-cresol, 4-hydroxybenzoate, and benzoate (Figure 1, Additional file 1). As with *G. metallireducens*, these compounds are predicted to be funneled to the central intermediate of benzoyl-CoA. In *Geobacter *strain FRC-32, there were also very similar orthologs to Dch, Had, Oah, and the fatty acid proteins that had higher transcript levels during benzoate oxidation in *G. metallireducens*, indicating that the dearomatized intermediate could also be oxidized to 3-hydroxypimelyl-CoA then acetyl-CoA (Figure 1, Additional file 1).

**Figure 8:**
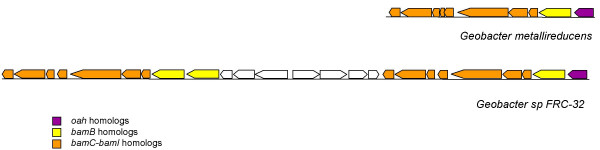
**The *oah *and *bamB*-*bamI *cluster in *G. metallireducens *and *Geobacter *strain FRC-32**. Schematic shows the duplication of this region in *Geobacter *strain FRC-32. Shown are *G. metallireducens *genes Gmet_2078-2088 and *Geobacter *strain FRC-32 genes NCBI accession numbers 110599026 – 110599050. Only those genes predicted to be involved in aromatics metabolism are shown in color.

Like *G. metallireducens*, there were no proteins similar to known benzoyl-CoA reductases encoded in the *Geobacter *strain FRC-32 genome. However, the *bamB*-*bamI *cluster, which was the most highly induced cluster of genes in the *G. metallireducens *genome during benzoate oxidation, and which was previously suggested as a candidate dearomatization enzyme, was duplicated in *Geobacter *strain FRC-32. Like *G. metallireducens*, *Geobacter *strain FRC-32 contains a cluster that encodes an Oah homolog, BamB, and the BamC-BamI genes (Figure 8). However, in *Geobacter *strain FRC-32, eight genes downstream of this cluster are two *bamB *homologs and a second, identically organized *bamC-bamI *cluster (Figure 8).

## Conclusion

The pathways of aromatics degradation in the Fe(III)-reducing, obligate anaerobe *G. metallireducens *appear to be similar to those from nitrate-reducing organisms that use more favorable electron acceptors. The genes are more closely related to those that have been studied in the nitrate-reducing *beta Proteobacteria *than to the nitrate-reducing or photosynthetic *alpha Proteobacteria *species. Toluene, phenol, *p*-cresol, 4-hydroxybenzaldehyde, 4-hydroxybenzoate, benzyl alcohol, benzaldehyde, and benzoate are all predicted to be channeled to the central intermediate benzoyl-CoA. De-aromatized products are predicted to be oxidized to 3-hydroxypimelyl-CoA.

However, there is one notable exception to this similarity. The reaction which has been shown to be the most energetically expensive in nitrate-reducing organisms, and which has been hypothesized to be different in obligate anaerobes, must be different in *G. metallireducens*. The completion of the genome sequence proves that *G. metallireducens *encodes no classical benzoyl-CoA reductase, the ATP-dependent enzyme that catalyzes the breaking of the aromatic bonds in benzoyl-CoA. Phylogenetic analysis, subunit content, and expression studies contradict the previous report that a benzoyl-CoA reductase was present in *G. metallireducens*, and support the previous report that no benzoyl-CoA reductase activity was found in *G. metallireducens *metabolizing benzoate.

The enzyme that dearomatizes benzoyl-CoA in *G. metallireducens *remains unknown. The 30-fold increase in transcript levels of the *bamB*-*bamI *cluster, and its phylogenetic relationship to genes in a benzoate-fermenter and in a field-isolate, support a role for these genes in benzoate oxidation. However, the lack of sequence similarity of these genes to characterized enzymes, and the discovery of highly induced paralogs in the genome indicate that further analysis of both sets of genes is necessary. Regardless of the mechanism, the dearomatization of benzoyl-CoA remains the most difficult step of the pathway, and *G. metallireducens *may offset energetic costs of this step by changing the way the acetyl-CoA produced by the pathway is oxidized.

The aromatics genes are organized in *G. metallireducens *in an island that shows substantial evidence of potential genetic mobility, and they are not found in closely related *Geobacteraceae *species that do not oxidize aromatic compounds. However, no evidence that these aromatics genes were recently transferred into the genome was found. The enzymes in the pathway up to the last step before fatty acid oxidation appear to be most similar to *Thauera*-like species, but the genes for fatty acid and TCA cycle oxidation, and the *bamB*-*bamI *cluster genes, though nearby, are more closely related to other organisms. Perhaps the genes were assembled in *G. metallireducens *from existing functional units into a new combination, as has been suggested for other catabolic pathways [55,56]. The continued effort to sequence other environmentally relevant *Geobacteraceae *species should allow a more detailed reconstruction of the evolutionary history of the genes.

The motivation for analyzing the genes of aromatics metabolism in *G. metallireducens *is to better understand *in situ *bioremediation by bacteria [57]. Similarity in the genes for aromatics degradation between *G. metallireducens *and the *Geobacter *species isolated from a toluene-contaminated bioremediation site suggests that the analyses presented here may be applied more broadly to the *Geobacter *community that predominates during bioremediation. A method for quantifying gene transcript levels in *Geobacter *species in the subsurface was recently developed [58,59], but has yet to be applied to the metabolism of aromatics. Hopefully, these results provide candidates for this type of study, to help clarify the physiological state and metabolic rates of the *in situ *species during bioremediation.

## Methods

### Genome analysis and annotation of genes

The complete *G. metallireducens *genome sequence was acquired from NCBI (accession NC_007517) [24]. Refinements of the automatically generated functional predictions for genes putatively involved in aromatics metabolism were made. Most similar sequences in other genomes were identified using BLASTp [60] with the non-redundant protein database [61] and UniProt [62]. Identification of functional domains and motifs was made using InterPro [63]. Transmembrane motifs and signal sequences were predicted with PsortB [64]. Comparisons to characterized proteins from aromatics metabolism were made with BLASTp [60] and alignments were made with ClustalX [65]. Phylogenetic relationships were inferred by distance based analysis using the neighbor joining method [66] in the Mega program [67]. Orthologs were defined as reciprocal best matches from BLASTp between two genomes that aligned over at least 60% of their length [68,69]. Total GC content, first codon position GC content and third codon position GC content were determined for each ORF in the genome using the cusp program in EMBOSS [70]. Effective number of codons (Nc) used was determined for each ORF in the genome using the chips program in EMBOSS [70]. Dinucleotide relative abundance difference with respect to the genome average was calculated using delta-rho [71]. GC content and GC skew (G-C/G+C) were calculated in a window of 20 kb with a step size of 5 kb.

### Cell culturing

*G. metallireducens *from our laboratory culture collection was cultured at 30°C in electron donor limited chemostats as previously described [72]. The dilution rate was 0.05 h^-1 ^with acetate (5 mM) or benzoate (1.25 mM) provided as the electron donor and ferric citrate (55 mM) as the electron acceptor. Acetate concentrations were measured with HPLC (Bio-Rad Aminex HPX-87H column), Fe(II) concentrations were measured by ferrozine assay, and protein concentrations were measured by bicinchoninic acid assay, all as previously described [72]. During growth with acetate, Fe(II) was 37.83 ± 1.88 mM, protein was 0.030 ± 0.005 mg/mL, and acetate was below detection limit (50 uM). During growth with benzoate, Fe(II) was 34.45 ± 6.58 mM and protein was 0.023 ± 0.002 mg/mL. Cells were harvested from three replicate chemostats for each of the electron donors.

### Gene expression analysis

RNA extraction, purification, and labeling were performed independently on each cell sample, as previously described [73]. Total cellular RNA from each replicate was isolated using TRIZOL (Invitrogen) and purified using RNeasy Mini Kit (Qiagen) with on-column DNase digestion performed with RNase-free DNase Set (Qiagen). 10 μg of purified total RNA was labeled as previously described [74], using random hexamers (Invitrogen) for priming and either Cy3-dUTP or Cy5-dUTP as the fluorophor. After labeling, RNA was removed by NaOH treatment and cDNA was purified with a PCR purification kit (Qiagen). The labeling efficiency was monitored by measuring the absorbance at 260 nm (for DNA concentration), 550 nm (for Cy3), or 650 nm (for Cy5). Two samples of each total RNA preparation were labeled, one with Cy3-dUTP and another with Cy5-dUTP for microarray hybridization.

DNA microarrays covering 3434 of the 3530 annotated protein-coding sequences of the completed *G. metallireducens *genome were constructed with 70mer oligonucleotide probes, designed as previously described [73,75]. All designed oligonucleotides were commercially synthesized without modification by MWG Biotech Inc. (High Point, NC). Oligonucleotides were adjusted to 100 pmol/μl in 50% DMSO, and two replicates per probe were spotted onto UltraGAPS glass slides (Corning Life Science, NY) using a Microgrid II robotic arrayer (Genomic Solutions Inc., MI) and fixed by UV cross-linking (600 mJ). Additionally, 6 concentrations (5, 25, 50, 100, 200, and 300 ng/μl) of genomic DNA were spotted (8 duplicates of each concentration per slide) as positive controls.

Microarray hybridization, washing, and scanning were carried out as previously described [73]. Cy5-dUTP-labeled cDNA targets from one benzoate culture were mixed with the Cy3-dUTP-labeled cDNA targets from one acetate culture and vice versa (dye swap). Equal amounts of Cy3- or Cy5-labeled probes were mixed and incubated at 95-98°C for 5 min, centrifuged to collect condensation, kept at 50°C, and applied onto microarray slides. Hybridization was carried out in hybridization chambers (Corning Life Sciences, Corning, NY) at 45°C for 16–20 h. Microarray slides were washed according to the instructions by the manufacturer (Corning), and dried with N_2_. The fluorescence intensity of both the Cy5 and Cy3 fluorophores was scanned using the ScanArray Express microarray analysis system (Perkin Elmer, Boston, MA).

Signal intensities were determined using 16-bit TIFF scanned images and ImaGene version 6.0 (Biodiscovery, Marina Del Rey, Calif.) to quantify spot signal, spot quality, and background fluorescent intensities. Empty spots, poor spots, and negative spots were flagged according to instructions and removed in subsequent analysis, as previously described [76]. Lowess normalization was performed on each slide using GeneSpring version 5.1 (Silicon Genetics, Redwood City, Calif.), and the results for the triplicate cultures were used for statistical analysis. Student *t*-test was used to calculate a *p*-value to test the null hypothesis that the expression level was unchanged for each spot. *P *values equal to or less than 0.01 were considered significant. Results were submitted to NCBI Gene Expression Omnibus, accession GSE5401.

## Authors' contributions

JEB carried out the analysis of genomes, analysis of gene expression data, and drafted the manuscript. QH carried out the gene expression studies. KPN carried out cell culturing. ZH participated in the gene expression studies. JZ participated in the study design and gene expression studies. DRL conceived of the study and helped to draft the manuscript. All authors read and approved the final manuscript.

## Supplementary Material

Additional file 1 ORFs in the aromatic island of the genome. For each of the 244 ORFs in the region the following is supplied: NCBI reference identification, abbreviated ORF identification used in the text, gene name, product, orthologous gene in the *Geobacter *strain FRC-32 genome, orthologous gene in the *Geobacter sulfurreducens *genome, the genus and species name and the NCBI reference number of the closest homolog in the non-redundant database, the start and end of the ORF in the *G. metallireducens *genome, the total GC content of the ORF, the GC content of each of the three codon positions, and the effective number of codons (Nc) used in the ORF. Click here for file

Additional file 2 Microarray experiment results. The ORFs from the whole *G. metallireducens *genome that had at least a 2 fold-change change of expression, with a p-value of at least 0.01. (NCBI Gene Expression Omnibus, accession GSE5401) For each of the 142 ORFs the following is supplied: NCBI reference identification, abbreviated ORF identification used in the text, gene name, product, fold change expression (benzoate/acetate), t-test P-value. Click here for file
